# Evaluation of a Screening Instrument for Autism Spectrum Disorders in Prisoners

**DOI:** 10.1371/journal.pone.0036078

**Published:** 2012-05-25

**Authors:** Louise Robinson, Michael D. Spencer, Lindsay D. G. Thomson, Andrew C. Stanfield, David G. C. Owens, Jeremy Hall, Eve C. Johnstone

**Affiliations:** 1 Division of Psychiatry, School of Molecular and Clinical Medicine, University of Edinburgh, Edinburgh, United Kingdom; 2 Department of Psychiatry, University of Cambridge, Cambridge, United Kingdom; 3 Patrick Wild Centre, University of Edinburgh, Edinburgh, United Kingdom; King's College London, United Kingdom

## Abstract

There have been concerns that individuals with autism spectrum disorders (ASDs) are over-represented but not recognised in prison populations. A screening tool for ASDs in prisons has therefore been developed.

**Aims:**

We aimed to evaluate this tool in Scottish prisoners by comparing scores with standard measures of autistic traits (Autism Quotient (AQ)), neurodevelopmental history (Asperger Syndrome (and High-Functioning Autism) Diagnostic Interview (ASDI)), and social cognition (Ekman 60 Faces test).

**Methods:**

Prison officers across all 12 publicly-run closed prisons in Scotland assessed convicted prisoners using the screening tool. This sample included male and female prisoners and both adult and young offenders. Prisoners with high scores, along with an equal number of age and sex-matched controls, were invited to take part in interviews. Prisoners' relatives were contacted to complete a neurodevelopmental assessment.

**Results:**

2458 prisoners were screened using the tool, and 4% scored above the cut-off. 126 prisoners were further assessed using standardised measures. 7 of those 126 assessed scored 32 or above (cut-off) on the AQ. 44 interviews were completed with prisoners' relatives, no prisoner reached the cut-off score on the ASDI. Scores on the screening tool correlated significantly with AQ and ASDI scores, and not with the Ekman 60 Faces Test or IQ. Sensitivity was 28.6% and specificity 75.6%; AUC was 59.6%.

**Conclusions:**

Although this screening tool measures autistic traits in this population, sensitivity for scores of 32 or above on the AQ is poor. We consider that this limits its usefulness and do not recommend that the tool is routinely used to screen for ASDs in prisons.

## Introduction

Autism spectrum disorders (ASDs), which include autism, Asperger syndrome and Pervasive Developmental disorder - Not Otherwise Specified, encompass impairments in social interaction, abnormalities in communication, and restricted, repetitive and stereotyped patterns of behaviour [1]. Results of prevalence studies vary, but community prevalence in adults in England is estimated to be 9.8 per thousand [2].

There is no evidence that individuals with ASDs are more likely to offend than those without [3]. However, the relatively high levels of ASDs found in high-security psychiatric settings [4], [5], have led to concerns that individuals with ASDs are not being recognised in the criminal justice system. Without such recognition, it may be difficult to make sense of their offence and assess criminal responsibility in order to allow an appropriate defence. While in prison these individuals may be particularly vulnerable to bullying or exploitation [6]. They are at increased risk of psychiatric co-morbidity, particularly ADHD and mood disorders [7], [8]. In addition, they may present management problems as a consequence of poor social and communication skills. Their early identification in prison would allow appropriate care to be provided, and risk of future offending to be more effectively assessed and managed.

The prevalence rate of ASDs in prisons is not known. A study asking staff in the Scottish Prison Service how many cases they were aware of yielded 19 people with an established diagnosis of learning disability and/or ASDs across 16 prisons [9]. This did not take into account undiagnosed cases or those where the diagnosis was not known to staff, and was not intended as a measure of prevalence.

In community samples, reported rates of ASDs vary. A rate of 15% was found for pervasive developmental disorder in a sample of young offenders referred for forensic psychiatric assessment in Stockholm [10]. A UK community study, although not a prevalence study, found lower rates of offending in individuals with a diagnosis of ASD than in a comparison group [11].

Diagnosis of ASDs usually requires a neurodevelopmental history and a clinical assessment. Although a number of clinical diagnostic instruments, such as the Autism Diagnostic Observation Schedule (ADOS) [12], are available, such instruments are too lengthy to be employed across a large population in a prison setting. Screening tools for other mental disorders have been used in prisons [13]. However, there is no such tool available for ASDs.

Against this background, a screening instrument for use in prisons has been devised. We sought to evaluate the screening questionnaire by comparing it against two other assessments used commonly by mental health professionals to assess for ASDs and an objective measure of social cognition, known to be impaired in individuals with ASDs [14].

## Methods

### Screening of the prison population

All 12 publicly-operated closed prisons in Scotland were invited to take part in the study. Prison officers completed the screening tool on convicted prisoners whom they had known for at least a week, during a specified one-week period.

The screening tool (Table 1) was designed by a group of researchers in the field of autism [15] in association with the charity Research Autism (www.researchautism.net), and based upon the Asperger Syndrome (and High-Functioning Autism) Diagnostic Interview (ASDI) [16]. It was intended to be completed for each prisoner by a prison officer who knows that prisoner well. Responses are based on behaviours that the officer will have observed as part of their professional role. No training is required to use the instrument and it takes on average 1.5 minutes to complete. A score of 5 or above was chosen as a positive score at the time of its design.

**Table 1:** ASD screening instrument.

Q		ASDI Area	Yes	Maybe	No
1	Appears ‘odd’ when compared to other prisoners of a similar age	1			
2	Described as a ‘loner’	1			
3	Appears reluctant to mix with other prisoners (e.g. during association periods). Keeps self to self	1			
4	Stands too close to other people (invades personal space) **and** seems oblivious of this	1			
5	When compared to other prisoners lacks a sense of humour or humour is regarded as odd. Doesn't seem to ‘get’ jokes	1			
6	Unusual gaze – stares **or** avoids eye contact	5			
7	Talks a lot about a narrow range of topics (regardless of interest of listener)	2			
8	May be comfortable talking with one person but uncomfortable or inappropriate in groups	1			
9	Asks the same question(s) over and over again (regardless of answers). Repetitive	2			
10	Good memory/ ability for facts or figures or very knowledgeable about a particular topic	2			
11	Popular with other prisoners. A ringleader (has a number of followers)	1			
12	Does not appear to follow conversations or instructions **or** frequently misunderstands them (e.g. – picks up on isolated words or may take what is said literally)	4			
13	Stickler for the rules- becomes upset if rules are broken or promises are not kept **(to an unusual degree)**	3			
14	Resists changes in routine – or is upset by them **(to an unusual degree)**	3			
15	Frequently interrupts or ‘talks over’ people	5			
16	Voice too loud or has a peculiar pitch **– or** speaks in a monotonous voice	4			
17	Tries to be sociable but is only ‘tolerated’ **or even** rejected by others	1			
18	Not keen on games involving physical exercise. (e.g. may avoid ball games or is poorly coordinated and very bad at them e.g. pool, football.)	6			
19	Clumsy, bumps into things or finds it difficult to walk or run in a straight line. Has problems keeping up or in step with others	6			
20	Complains about noise or bright lights	n/a			

### Interviews

Following the screening process, we aimed to interview and further assess all prisoners scoring above the proposed cut-off of 5 on the screening tool, and an equal number of age and sex-matched controls scoring below 5. However, as very few prisoners scored above 5, we invited all prisoners scoring above 0 to participate in interviews, along with age and sex-matched controls (scoring 0).

Interviews and assessments with prisoners were carried out by a team of psychiatrists trained in the measures used. Interviewers were blind to screening status. Participants in whom the initial clinical assessment suggested possible current mental disorder were fully clinically screened with a standardised instrument, the Clinical Interview Schedule [17]. All interviewed prisoners were asked to consent for a relative to be contacted in order to conduct a telephone interview.

Background information was obtained from all interviewed participants, including age, date of admission and estimated date of liberation from prison. Forensic, substance misuse, past medical and psychiatric, educational and employment histories were taken. Participants provided accounts of past offending. Current IQ was measured using the Quick Test [18], a brief, standardised measure of intelligence that can be used in non-readers; and reading age using the Schonell Graded Word Reading Test [19].

Three standardised measures were used with the interviewed group of prisoners- a measure of autistic traits (Adult Autism Spectrum Quotient [20]); an interview with a relative (Asperger Syndrome (and High-Functioning Autism) Diagnostic Interview [16]); and a measure of facial emotion recognition (Ekman 60 Faces Test [21]).

#### Adult Autism Spectrum Quotient (AQ)

The AQ is a self-report questionnaire that measures a range of mild autistic traits in a relatively brief and simple format. An initial study demonstrated excellent sensitivity and specificity in the identification of participants with ASDs [20]. In the general population, 80% of adults of normal intelligence meeting criteria for ASDs would be expected to score 32 or above in the test, in comparison with 2% of controls. The AQ was not devised specifically for antisocial groups, and some of the questions refer to aspects of life unfamiliar to many prisoners, such as visits to theatres and museums. However, good sensitivity and specificity in identifying individuals with ASDs has been demonstrated in a forensic psychiatric sample [22]. Due to low literacy levels in the current study population each question was read aloud to the participant.

#### Ekman 60 Faces Test

This neuropsychological test of basic facial emotion recognition consists of a battery of photographs of faces drawn from the Ekman and Friesen series [21]. Sixty photographs, comprising ten representing each of six basic emotions (happiness, surprise, disgust, fear, anger and sadness) are separately displayed upon a computer screen in a pseudo-random order. The participant is required to identify which of the six emotions each photograph represents. This test has been used successfully to characterise deficits in emotion recognition displayed by adults with ASDs [14].

#### Asperger Syndrome (and High-Functioning Autism) Diagnostic Interview (ASDI)

This structured clinical interview was developed to include a range of aspects of behaviour typically affected by ASDs [23]. It is designed for use with a first-degree relative who has known the individual well since their childhood. Relatives of prisoners who had provided consent were contacted. The ASDI was carried out by telephone by the same researcher (LR), blind to screening status.

### Ethics Statement

This study was approved by the Scottish Prison Service Ethics Committee and the Multicentre Research Ethics Committee (MREC). Written information about the study was displayed in areas agreed with individual prisons, and prisoners could choose to opt out of the screening process. Before participation in interviews, prisoners were given written information and written informed consent was obtained. MREC stipulated that only convicted prisoners could be included in the study. Patients' relatives were provided with written information, and either written (where possible) or verbal consent was obtained from them, documented, dated and signed by the researcher. Verbal consent was used both because of anticipated problems with literacy and the likelihood of the lifestyles of some individuals leading to difficulty in receiving and returning forms by mail. This was approved by MREC.

### Statistical Methods

Data from prison officers, prisoners and relatives were analysed anonymously using SPSS 14.0 for Windows.

## Results

Screening, interviews and assessments took place between February 2008 and September 2009.

### Screening Tool

2458 convicted prisoners were screened using the tool. The convicted prisoner population at that time was 6156 [24], therefore approximately 40% of the convicted population in Scotland was screened. Prisons included local and long-stay prisons, one male Young Offenders' Institution (YOI) and one women's prison and YOI. 127 of the prisoners screened were women. 15 prisoners from Inverness were screened at the health centre; all other prisoners were screened by staff on the prison halls (main living areas).

**Figure 1:**
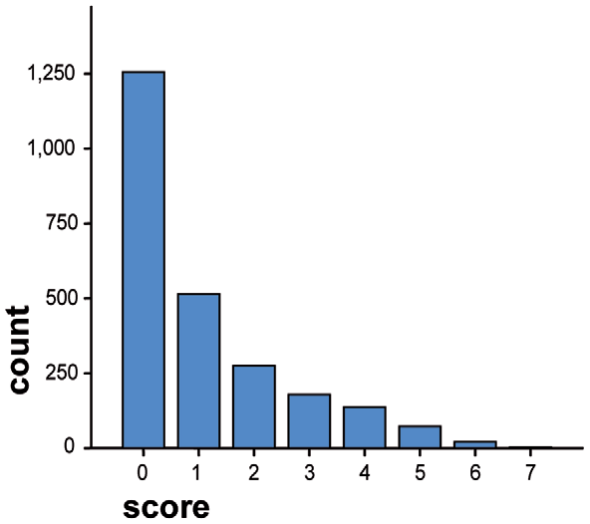
Distribution of scores on the screening tool on all prisoners screened (n = 2458).

Minimum score on the tool was 0, maximum was 7. Median score across all prisons was 0, (interquartile range 0–2) (Figure 1).

Median score from those prisoners attending Inverness prison health centre was 4 (n = 15, IQ range 2–4). When those from the health centre in Inverness were excluded from the total sample of prisoners screened, median score remained 0 (0–2) (n = 2443). Distribution of scores across prisons is shown in Table 2. 97 prisoners (4.0%) scored 5 or more, the cut-off chosen for the screening tool at its design.

**Table 2:** Scores on screening tool by prison.

Prison (N)	Prisoner group	Median score (interquartile range)
Edinburgh (340)	Local, male, all sentence lengths	2 (1–4)
Barlinnie (574)	Male, all categories	1 (0–3)
Perth (143)	Male, short and long-term	0 (0–1)
Shotts (371)	Male, long-term	1(0–2)
Greenock (61)	Male, short-term and long-term	2 (0–4)
Dumfries (121)	Male, short-term and offence-related protection prisoners	1 (0–1)
Peterhead (280)	Male, long-term sex offenders	1 (0–1)
Polmont (226)	Male young offenders (16–21)	0 (0–1)
Cornton Vale (127)	Female, young offenders and adult, all categories	1 (0–2)
Aberdeen (113)	Local, male up to 4 years	0 (0–1)
Inverness (67)	Local, male, short-term	0 (0–1)
Glenochil (35)	Male, long-term	0 (0–2)
Total 2458		

On comparison of the distribution of scores between prisons, the Kruskall –Wallis one way analysis of variance test is significant beyond the .01 level: chi-square (11) = 197.97; p<.01, meaning that there are statistically significant differences between the prisons.

#### Reliability

Data on reliability were obtained from HMP Peterhead only. Data on inter-rater reliability data were not obtained. Regarding intra-rater reliability nine prisoners were re-scored after a week had elapsed by the officer who had first scored them. Median score for the 9 prisoners for the first screen was 0, (IQ range 0–2), and for the repeat screen was 2 (IQ range 1–4.5). There was no significant correlation over time between the ratings of the same prison officer for the same subject (ICC<0), and intra-rater reliability was therefore poor.

### Interviews

103 participants scoring above zero on the screen were invited for interview along with an equal number of age and sex-matched participants scoring zero. 51 of the 103 (49.5%) participated, of whom 33 had scored 5 or above on the screen (the cut-off).27 refused (26%), and 17 (16.5%) were unavailable (at court, liberated or transferred). For one individual who did not attend the reason was not known. 76 (73.7%) of those invited and scoring zero on the tool chose to participate. In total, 127 prisoners who had been scored with the screening tool attended for interview, and 126 took part in all of the further assessments. Seven of those interviewed were women.

### Participant Characteristics

#### IQ/ reading age

Age, IQ and reading age are shown in Table 3. On the Quick Test one participant's score was too low to allow calculation of IQ. IQ was estimated at less than 70 in 6 participants.

**Table 3:** Demographic characteristics of participants.

	N	Mean; (range, standard deviation)
**Age (years)**	126	35.2 (17.7–65.7; 11.3)
**IQ**	125	92.5 (45–130; 15.4)
**Reading Age (years)**	125	12.6 (6.8–15; sd 1.8)

#### Health/Substance Use

Mean estimated alcohol intake in the week before prison admission was 91.1 units per person (males 91.5; females 83.4) (range 0–595, sd 123.2). 102 (81%) participants had ever used illegal drugs, and 46 (36.5%) had used drugs while in prison. 42 (33%) had a history of IV drug abuse. 69 (54.8%) had a history of head injury leading to hospital admission or loss of consciousness. 74 (58.7%) were being prescribed medication, 22 of whom were prescribed methadone. 77 (61.1%) had ever seen a psychiatrist, 17 (13.5%) stated that they had been detained under the Mental Health Act. Six said that they had been given diagnoses of schizophrenia or psychosis, 13 depression, 6 substance misuse problems, 5 PTSD, 6 ADHD, and one possible ASD. Two had been seen for anger management, and 43 gave a history of deliberate self harm.

#### Forensic Characteristics

See Table 4. 114 (90.5%) prisoners had previous convictions and 94 (74.6%) had served previous prison sentences.

**Table 4:** Self-reported index offence.

Offence Type	N (%)
**Violent**	86 (68.3)
***of which sexual***	22 (17.5)
**Drug-related**	16 (12.7)
**Theft**	9 (7.1)
**Breach of the Peace**	5 (4.0)
**Other**	10 (8.0)
**Total**	126 (100)

#### Education/Employment

36 (28.6%) of prisoners had received special educational support at school. 114 (90.5%) said that they can read and write. 85 (67.5%) had been excluded from school, and 47 (37.3%) had formal educational qualifications. 107 (84.8%) had ever been employed.

#### Mental Illness Screen

Seven prisoners were examined with a formal mental illness screen [17]. Three had no symptoms, two had symptoms of depression and anxiety, one had dissociative symptoms, and one had symptoms suggestive of an organic brain syndrome.

#### Autism Quotient

**Figure 2:**
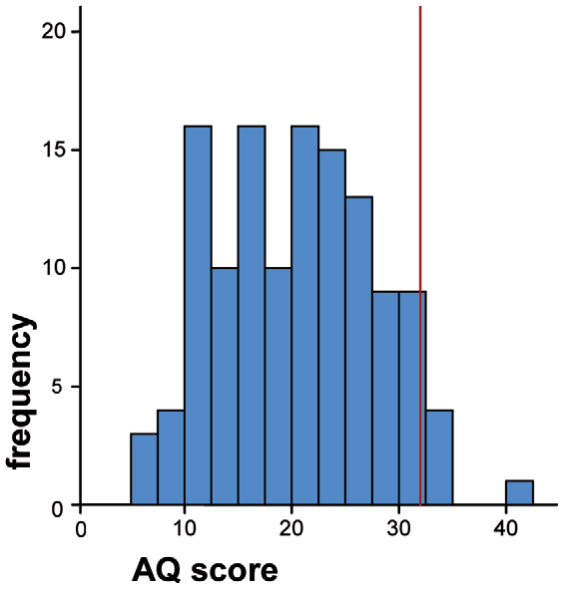
Distribution of AQ scores, showing cut-off of 32.

Mean AQ score was 20.1 (range 6–41, sd 7.3) (Figure 2). Seven of the 126 participants (5.65%) scored 32 or above, the cut-off at which further investigations for ASDs are recommended by the authors [20].

#### ASDI

**Figure 3:**
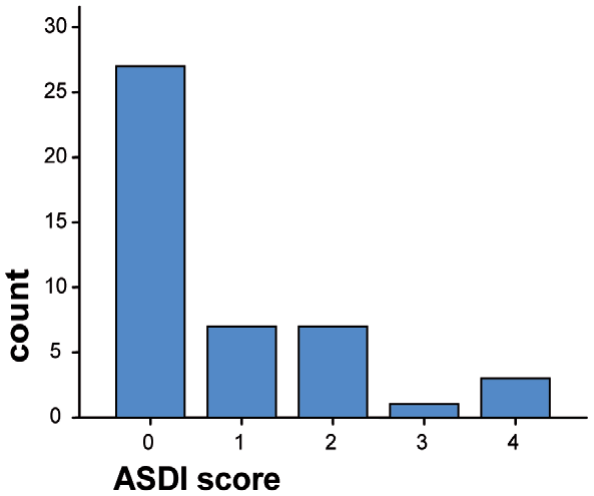
Score distribution on the ASDI.

An ASDI was carried out with 44 prisoners' relatives (3 female and 41 male prisoners). No participant reached the diagnostic cut-off score of 5 (median score was 0, interquartile range 0–1.75) (Figure 3).

#### Ekman 60 Faces Test

This test provides a score out of 10 for each of the 6 emotions (happiness, sadness, disgust, fear, anger, surprise) and a total score out of 60. 126 screened prisoners were examined, mean score was 41.1 (range 24–55, sd 7.3). Performance was not consistent across emotion type, with prisoners performing best at recognising happiness (mean score 9.8) and worst at fear (mean score 4.2). The prisoner group performed poorly at this task in comparison with normal IQ- and sex-matched controls [25] .

### Relationship between measures

AQ and ASDI scores (rho = 0.35, p = 0.018), and AQ and IQ scores (rho = 0.25, p = 0.006), showed significant correlations. IQ and Ekman score were also significantly correlated (rho = 0.35, p<0.001). There was no significant association between IQ and ASDI score. While AQ and Ekman scores showed a significant correlation (rho = 0.25, p = 0.005), this becomes non-significant when IQ is used as a covariant.

### Relationship between measures and screening tool scores

#### AQ and ASDI

**Figure 4:**
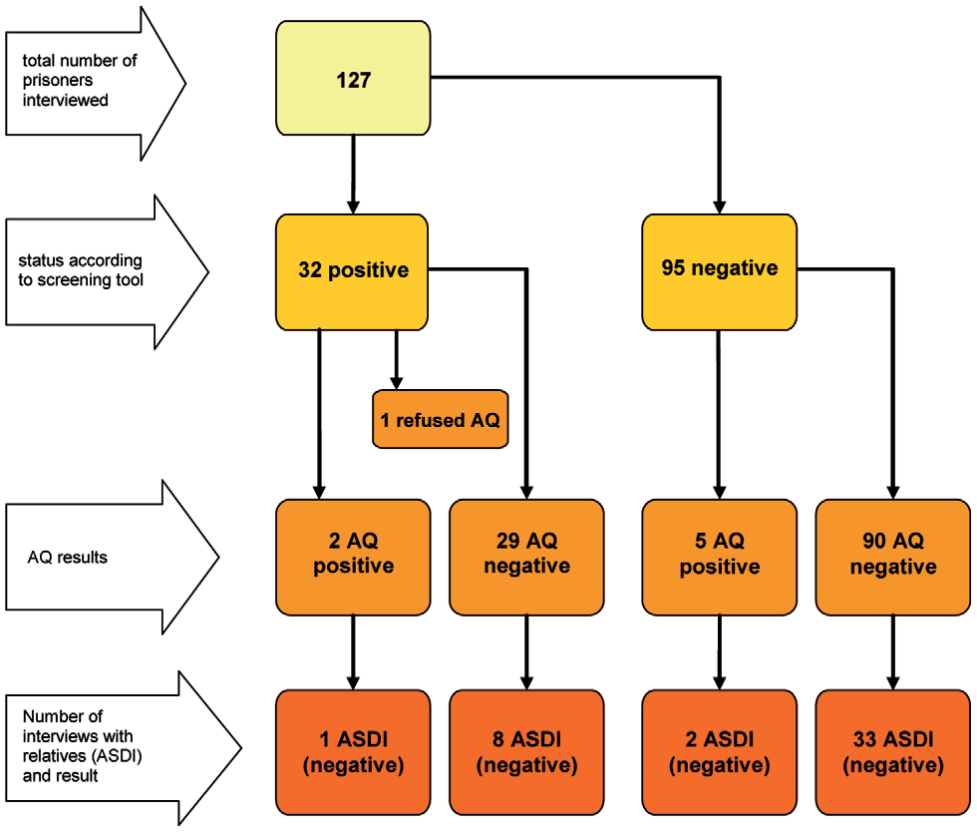
Summary of screening tool, AQ and ASDI results.

Figure 4 shows the relationship between categories of screening tool status (above or below the cut-off of 5), AQ status (above or below 32) and ASDI status (above or below 5).

A statistically significant association was found between the numerical scores on the screening tool and AQ score (rho = 0.177, p = 0.047). A statistically significant correlation was also found between the screening tool score and ASDI (rho = 0.37, p = 0.012).

#### Relationship with Ekman 60 test scores

There was no statistically significant correlation between score on the screening tool and Ekman 60 score (rho = 0.21, p = 0.41).

#### Relationship with IQ

The screening tool score did not correlate significantly with IQ (rho = 0.05, p = 0.579). In addition, there was no significant association between the screening tool score and reading age or whether an ASDI was performed.

### Characteristics of the screening tool

In the tool design, a score of 5 was designated as the cut-off (i.e. individuals scoring 5 or above were screened as positive).

#### Comparison against AQ results

In this analysis a score of 32 or above on the AQ represents a case. The rate of a score of 32 or above was 5.5% in this sample. We note, however that the AQ is not a diagnostic instrument and that all three participants scoring 32 or above on the AQ who were also assessed using the ASDI did not reach the diagnostic threshold on that measure.

Table 5 shows the contingency table for screening tool cut off against AQ cut-off (chi-square = 0.063, p = 0.80).

**Table 5:** Contingency table: screening tool results and AQ cut off.

		AQ cut off reached (case)	AQ cut off not reached	Total
**5 or above on screen**	**yes**	2	29	31
	**no**	5	90	95
**Total**		7	119	126

#### Sensitivity and specificity of the screening instrument

**Figure 5:**
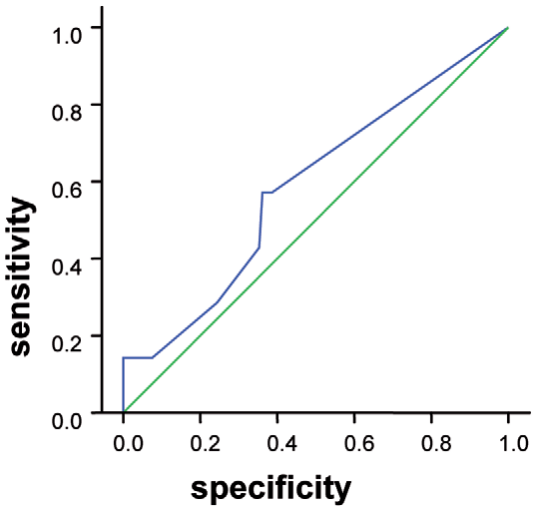
ROC curve demonstrating sensitivity and specificity.

Sensitivity was 28.6% and specificity 75.6%. A ROC curve was plotted (Figure 5). Area under the curve is 59.6% (where a figure close to 100% suggests a good screening measure and a figure of 50% indicates that it is no better than chance); significance is 0.44, i.e. probability that the test performs better than at random is low. Regardless of cut-off score chosen, sensitivity in particular is low (Table 6.).

**Table 6:** Sensitivity and specificity of the screening tool.

Screen score	Sensitivity	Specificity
0	1.0	0.00
1	0.57	0.61
2	0.57	0.62
3	0.57	0.62
4	0.43	0.65
5	0.29	0.76
6	0.14	0.92
7	0.14	1.00

## Discussion

This study examined a tool designed to be completed by prison officers with the aim of screening for ASDs in prisoners.

### Generalisability

The tool was evaluated in a large sample of convicted prisoners that included both sexes and range of ages. Scores on the Quick Test can be compared with those obtained during a survey of the prison population of England and Wales [26]. In that sample 24% of male sentenced and 16% of female sentenced prisoners scored 41 or more (equivalent to an IQ of 100 or more), while in the current, Scottish sample 40% of males and 29% of females scored 41 or more. The sample in this study therefore appears to have a relatively larger proportion of individuals with an IQ score above 100, although it does not reach the expected population rate of 50%. With respect to low IQ, a study of both remand and sentenced prisoners in England and Wales [27] found that 4% scored 25 or less on the Quick Test (indicating an IQ of less than or equal to 65) and also had no educational qualifications. Similarly, in the current sample, 6 prisoners (4.7%) met both of these criteria. The high levels of substance misuse and head injury in this sample are in also keeping with other prison populations [28] [29].

Although there was considerable past psychiatric contact, we did not find evidence of high rates of current major mental illness. This contrasts with data from other sentenced prisoner populations. For example, rates of current psychotic illness have been estimated as 7% of male sentenced prisoners [26] [30], and 14% of female sentenced prisoners in England and Wales [26]. Results from this study are keeping with prevalence studies in remand populations which suggest that levels of major mental illness in prisons may be lower in Scotland, (2.3%), than in England and Wales (10%) [31] [26], most likely as a result of greater diversion from the prison system in Scotland [32].

### Importance of the test

This screening test does appear to measure autistic traits. Its results correlate both with a self-report measure of autistic traits (AQ) and scores on a structured relative interview (ASDI). Importantly, this relationship remains when we control for IQ. The facial emotion recognition (Ekman 60 Faces Test) scores do not correlate with measures of autistic traits and appear to reflect an ‘antisocial’ pattern of deficits discussed further elsewhere [33].

Although specificity is good, sensitivity against AQ scores is poor and, although limited, the data suggest poor reliability. The poor intra-rater reliability may relate to individual characteristics of this tool or reflect more general difficulties in a design using prison officers. We conclude therefore that although this tool is simple and practical, its use in a prison population is limited by its poor sensitivity and intra-rater reliability.

### Prevalence

This study was not designed to estimate ASD prevalence. However, it is to our knowledge the largest ever study examining screening for ASDs in a prison setting. We did not find large numbers of individuals with high self-report scores of autistic traits. In addition, no developmental history taken was suggestive of an ASD. This may be because individuals with ASDs did not take part in assessments (selection bias) or that the particular tools used did not identify individuals with ASDs in this population. However, it may be because levels of ASDs in this prison population are in fact low. This might be due to diversion of such individuals early in the criminal justice process, or because prisoners with ASDs may not tolerate a prison environment resulting in transfer to hospital once admitted to prison (these explanations could explain the relatively high rates of ASDs identified in the special hospitals). It is also possible that individuals with ASDs are less likely to offend, and therefore would be under-represented throughout the criminal justice system [11].

### Limitations

There are several limitations to this study. We were unable to examine remand prisoners. Remand prisoners differ from convicted populations and in particular are more highly morbid with respect to mental disorders [26]. However, although there may therefore have been more cases of ASD, we do not consider that the performance of the tool would have been affected and this would not therefore alter our conclusions.

Although most prisoners took part in the screening, fewer of those screening positive than negative on the tool chose to take part in the study. As we know that the screen does provide some measure of autistic traits, this may mean that individuals with ASDs were less likely to take part in the interviews. Again, we do not consider that this would have altered the overall assessment of the tool.

It did not prove possible to obtain data on inter-rater reliability, and data on intra-rater reliability were limited. We were reliant upon the co-operation of prison officers to obtain these, and reasons for the difficulties may have included constraints on their time or an inadequate explanation on our part to officers for the reasons for repeat screenings. These data are important, however. Those we do have suggest poor reliability. This suggests that the screen would be of limited use regardless of its other characteristics. Although it is unlikely that this screen will be used, this is an important consideration in the design of other screening tools completed by prison officers.

We did not attempt to provide a DSM-IV diagnosis of an ASD, and did not carry out the gold-standard test of a clinical assessment. Diagnosis of this condition is complex and particularly difficult in a prison environment, with its rapid turnover and frequent and unannounced movement of prisoners. It appears likely, therefore, that using a full clinical assessment would have led to lower numbers of participants in the study.

### Conclusions

To our knowledge, this is the largest study of a screening tool for ASDs in a prison carried out to date. Although specificity was good, the sensitivity of this tool was poor in this convicted Scottish prisoner population. We do not, therefore, recommend its use in screening for ASDs in prisons.

Although this was not a prevalence study, we did not find evidence to suggest that ASDs are common in this population. In addition, we did not find evidence suggesting elevated rates of current major mental illness in this population. However, we did find high levels of head injury and substance misuse. The extremely high self-reported levels of alcohol use in particular (average intake for men more than 4 times the recommended weekly limit, and for women almost six times) are a significant problem in this population. At present alcohol misuse is not routinely screened for in Scottish prisons and it is likely that many individuals with alcohol misuse disorders are not identified by prison staff [34].

We suggest that rather than routinely screen for ASDs in prison, staff should be encouraged to raise concerns about individuals struggling to cope in prison. We also recommend that mental health staff should be trained to recognise ASDs and that there should be access to specialist ASD services where clinically appropriate.
